# Response of *Escherichia coli* chemotaxis pathway to pyrimidine deoxyribonucleosides

**DOI:** 10.1128/spectrum.02048-25

**Published:** 2025-10-14

**Authors:** Malay Shah, Wenhao Xu, Victor Sourjik

**Affiliations:** 1 Max Planck Institute for Terrestrial Microbiology & Center for Synthetic Microbiology (SYNMIKRO), Marburg, Germany; South China Sea Institute of Oceanology, Chinese Academy of Sciences, Guangzhou, Guangdong, China

**Keywords:** bacteria, chemotaxis, motility, nucleotide metabolism, FRET, signal transduction

## Abstract

Nucleosides are essential components of all living cells. Bacteria use salvage pathways to import nucleosides from their environment and to utilize them for nucleic acid biosynthesis, but also as alternative sources of carbon, nitrogen and energy. Motile bacteria commonly show chemoattraction towards nutritionally valuable compounds, and in this work, we demonstrate that the chemotaxis pathway of *Escherichia coli* exhibits specific attractant response to pyrimidine nucleosides. The most sensitive response, in the sub-micromolar range, was observed for pyrimidine deoxyribonucleosides, thymidine (deoxythymidine) and deoxycytidine. In contrast, pyrimidine ribonucleosides elicited weaker and less sensitive response, and no response to pyrimidine nucleobases was observed in the micromolar range of concentrations. Furthermore, no response was observed to tested purine nucleosides or nucleobases. Our subsequent analysis revealed that the pathway response to pyrimidine deoxyribonucleosides is mediated by the minor *E. coli* chemoreceptor Tap, whereas the response to the ribonucleoside uridine relies on a different mechanism. The observed narrow dynamic range of this response indicates that sensing of deoxyribonucleosides is indirect, likely via an unknown periplasmic binding protein that interacts with Tap.

**IMPORTANCE:**

Chemotactic behavior is highly important for bacterial ecology, enabling motile bacteria to locate environments that are optimal for growth, and it became a paradigm for bacterial environmental sensing and signal transduction. However, even for model organisms, the spectrum of stimuli sensed by the chemotaxis pathway is not fully known, which limits our understanding of the physiological and ecological relevance of the chemotactic behavior. Here, we identified pyrimidine deoxyribonucleosides as a novel and highly specific class of chemoeffector metabolites for *Escherichia coli*, the most studied chemotaxis model. Our work expands the number of bacterial species that exhibit chemotactic responses to nucleotide derivatives, strengthening the notion that pyrimidines and purines constitute a highly important class of chemoeffectors for many bacteria.

## INTRODUCTION

Motile bacteria use chemotaxis to navigate chemical gradients in their environment, locating optimal niches for their proliferation and survival (1–3). The gradient navigation relies on the detection of temporal changes in ligand concentrations, to which bacteria respond by adjusting their rate of tumbling. The molecular details of this process are well understood (1, 4): Effectors are typically sensed by the transmembrane chemoreceptors that form a ternary complex with an adaptor protein CheW and a histidine kinase CheA. Attractant sensing induces a conformational change in chemoreceptors that inactivates the autophosphorylation activity of the receptor-associated CheA, thereby lowering phosphorylation of the response regulator CheY. Since phosphorylated CheY (CheY-P) interacts with the flagellar motor to induce cell tumbles, reduced levels of CheY phosphorylation result in prolonged periods of straight swimming up the gradients of attractants. The chemotaxis machinery of *E. coli* further includes the CheY-P phosphatase CheZ, as well as the adaptation module consisting of CheR and CheB that modulates receptor activity and sensitivity to ligands through methylation and demethylation of receptors on specific glutamate residues.

The chemotaxis pathway of *E. coli* is known to sense a number of nutrient compounds, including amino acids (5, 6), sugars (7, 8) and dipeptides (9), but also signaling molecules, such as indole (10–12), autoinducer 2 (11, 13, 14), and mammalian hormones (10, 15, 16). *E. coli* has four transmembrane chemoreceptors: two major receptors, Tar and Tsr, and two minor receptors, Trg and Tap (1, 4). Primary ligands of Tar and Tsr are amino acids aspartate and serine, respectively, that bind directly to their periplasmic sensory domains. Other chemoeffectors are sensed through the interaction between the receptor sensory domains and a ligand-bound periplasmic binding protein that is a component of an ATP binding cassette (ABC) transporter. Such indirect sensing has been established for maltose in the case of Tar (7), and for the autoinducer 2 in the case of Tsr (13). Indirect sensing is the only established mode of responses mediated by the minor receptors, including the responses of Trg to glucose, galactose, and ribose (17), and of Tap to dipeptides (9). Besides these well-established mechanisms, cytoplasmic regions of chemoreceptors can mediate responses to unconventional stimuli, such as phenol, temperature, or cytoplasmic pH (18), as well as to the substrates of the phosphotransferase system (PTS) (8), although these sensory modes remain poorly understood.

Amino acids and sugars are known chemoeffectors not only for *E. coli* but also for other bacteria, supporting the notion that bacteria are primarily attracted by nutrients (19). Another common class of important metabolites that serve as chemoeffectors are nucleobases and their derivatives (20–23). Indeed, like other bacteria, *E. coli* can uptake and salvage nucleobases and nucleosides from the environment to replenish its nucleotide pool but also to use them as alternative sources of carbon, nitrogen, and energy (24). A previous study using a capillary assay of bacterial chemotaxis has shown that *E. coli* exhibits a Tap-mediated chemoattraction to millimolar concentrations of pyrimidine nucleobases (25), but the mechanism and scope of this response was not further investigated. Here, we demonstrate that Tap mediates highly sensitive response to pyrimidine deoxyribonucleosides, thymidine (alternatively called deoxythymidine) and deoxycytidine, whereas no response to pyrimidine nucleobases thymine or cytosine was observed at tested micromolar concentrations. We therefore conclude that pyrimidine deoxyribonucleosides, rather than nucleobases (25), are specific chemoattractants for *E. coli*. Our results further suggest that this sensing of deoxyribonucleosides by Tap is indirect, likely mediated by a yet unknown periplasmic binding protein. *E. coli* also responded to pyrimidine ribonucleosides cytidine and uridine, but this response was less sensitive and, for uridine, apparently mediated by a Tap-independent mechanism.

## RESULTS AND DISCUSSION

### Characterization of *E. coli* response to nucleosides

**Fig 1:**
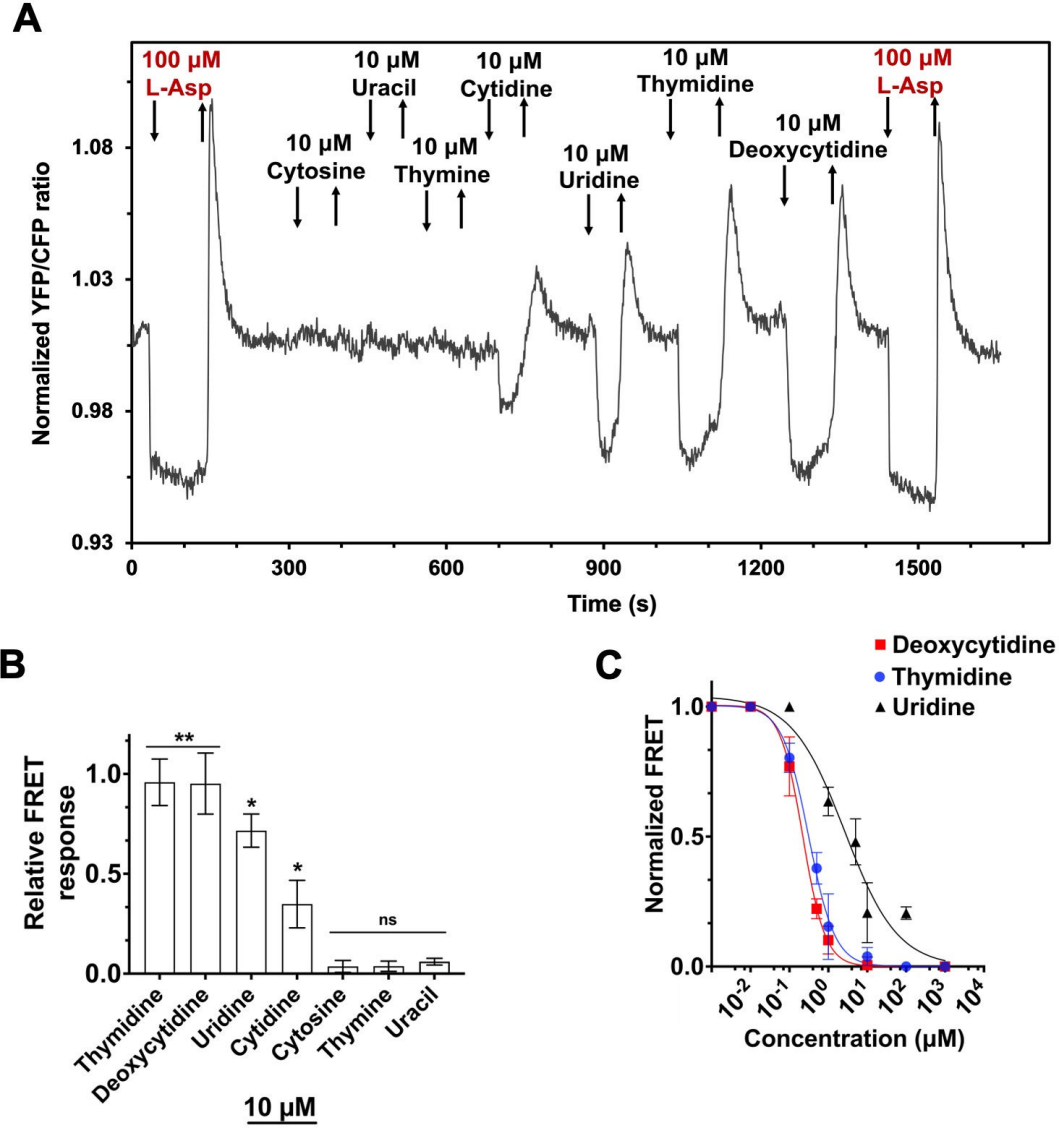
FRET measurements of the chemotaxis pathway response to pyrimidine nucleosides and nucleobases. (**A**) *E. coli* cells expressing CheZ-CFP/CheY-YFP FRET pair were immobilized in a flow chamber, adapted to tethering buffer and stimulated by addition and subsequent removal of 10 µM of indicated compounds. Saturating stimulation with 100 µM L-aspartate (L-Asp; red) was used as a control. FRET measurements for all figures were detrended as described in the Materials and Methods. FRET signal is represented by the ratio of YFP to CFP emission, normalized to the YFP/CFP ratio at the beginning of the measurement and compensated for the gradual drift in the baseline as described in the Materials and Methods. (**B**) Response amplitudes of buffer-adapted cells to 10 µM of indicated compounds, measured as the initial change in the YFP/CFP ratio after stimulation and normalized in each experiment to the response amplitude for 100 µM L-aspartate, and calculated from three biological replicates. Error bars indicate standard deviations. Significance of the response compared to zero was assessed using Student’s one-sample *t*-test (ns: non-significant, **P-value ≤ 0.01, *P-value ≤ 0.05). (**C**) Dose-response curves measured by FRET for indicated compounds. Cells were stimulated by step-like addition and subsequent removal of increasing amounts of an attractant, and the initial change in the YFP/CFP ratio was quantified. After each stimulation, cells were re-adapted to the buffer. The FRET response for each step was normalized to the maximal FRET response. Error bars indicate standard deviations, and means are derived from three biological replicates. Data were fit using the Hill equation, with EC_50_ fit values and respective 95% CI (shown in brackets) for each compound being 3.7 µM (2.3 µM to 5.6 µM) for uridine; 237 nM (200 nM to 280 nM) for deoxycytidine; 337 nM (277 nM to 400 nM) for thymidine.

To systematically characterize the response of *E. coli* chemotaxis pathway to pyrimidine nucleobases, ribonucleosides, and deoxyribonucleosides, we used an assay of the pathway activity based on the Förster resonance energy transfer (FRET) (26). For that, we expressed the pair of interacting pathway proteins fused to cyan and yellow fluorescent proteins, CheZ-CFP and CheY-YFP. Since the interaction of CheY with its phosphatase depends on phosphorylation, it allows real-time monitoring of the pathway activity in a cell population using FRET (26, 27). To test their response to different pyrimidines, *E. coli* cells expressing the FRET pair were immobilized in the flow chamber and exposed to different concentrations of tested compounds. Responses were recorded as a change in the ratio of the YFP to CFP fluorescence (FRET ratio), with L-aspartate (L-Asp), a canonical chemoattractant for *E. coli*, being used as a positive control. Since attractant inhibits CheA activity, leading to a reduction in the fraction of interacting CheY-YFP and CheZ-CFP proteins, addition of L-aspartate caused a rapid decrease in the FRET ratio (Fig. 1A). The response to 100 µM L-aspartate is known to completely inhibit the pathway activity, thus reducing the YFP to CFP ratio to the level observed in the absence of energy transfer (27). Subsequent removal of an attractant leads to an increase in the CheA activity with concomitant increase in the fraction of interacting FRET reporter proteins. The resulting pathway activity is initially higher than that in the adapted cells because the CheR-mediated adaptive changes in receptor methylation during prolonged stimulation with attractant increase receptor activity bias. This transient overshoot of the pathway activity upon removal of the chemoattractant is gradually reset to the steady-state level by the CheB-dependent receptor demethylation. The change in the normalized YFP to CFP ratio directly after the attractant stimulation and prior to any adaptive changes in the pathway activity was used to quantify the amplitude of the FRET response to tested compounds (Fig. 1B) and to construct the corresponding dose-response curves (Fig. 1C).

Our results demonstrate that, at the tested concentration of 10 µM, stimulation with pyrimidine nucleobases (cytosine, uracil, or thymine) displayed no measurable FRET response (Fig. 1A and B). In contrast, ribonucleosides (cytidine and uridine) elicited a clear attractant response, although at 10 µM, it was weaker than the maximal response to L-aspartate (Fig. 1A and B). The strongest and most sensitive response was observed upon stimulation with deoxyribonucleosides (deoxycytidine and thymidine), with a distinct change in FRET already at 300 nM (Fig. 1C; Fig. S1) and the amplitude of response being similar to the saturating stimulation with 100 µM L-aspartate already at 1 µM. In contrast, no response was observed upon stimulation with purine nucleobases or deoxyribonucleosides (Fig. S2).

To further compare the sensitivity of *E. coli* chemotaxis pathway to pyrimidine deoxyribonucleosides and ribonucleosides, we measured the dose-dependence of the FRET response to deoxycytidine, thymidine, and uridine. Whereas the half-maximal inhibition of the pathway activity by uridine was observed at EC_50_ of ~4 µM, the EC_50_ values for deoxycytidine and thymidine were 10-fold lower, at ~300 nM (Fig. 1C). Such sensitivity is within the same range as the EC_50_ values measured by the FRET assay for the established high-affinity *E. coli* attractants, including aspartate (193 nM) and serine (163 nM) that bind to their respective chemoreceptors directly, and galactose (15 nM), ribose (63 nM) and dipeptides (365 nM) that are sensed via periplasmic binding proteins (28). We thus conclude that pyrimidine deoxyribonucleosides are high-affinity chemoattractants for *E. coli*.

### Sensing of pyrimidine deoxyribonucleosides is likely indirect and mediated by Tap

To investigate whether the sensing of pyrimidine deoxyribonucleosides is direct or indirect, we next investigated the dynamic range of the chemotaxis pathway response. The dynamic range represents the most characteristic difference between chemoeffector ligands that directly bind to *E. coli* chemoreceptors, and those that rely on binding to periplasmic proteins to relay the signal to chemoreceptors (28). Directly binding ligands can be sensed over four to five orders of magnitude of ambient concentrations (5, 27), because the receptor methylation system enables efficient tuning not only of the pathway activity but also of the receptor sensitivity to these ligands as cells become exposed to their increasing concentrations. In contrast, while the response to indirectly binding ligands is similarly adaptive in respect to the pathway activity, binding of these ligands to their periplasmic binding proteins saturates and cannot be tuned by the adaptation system. As a consequence, the dynamic range of response to these ligands is much narrower than for directly binding ligands, spanning only two orders of magnitude of ligand concentrations (7, 28).

**Fig 2:**
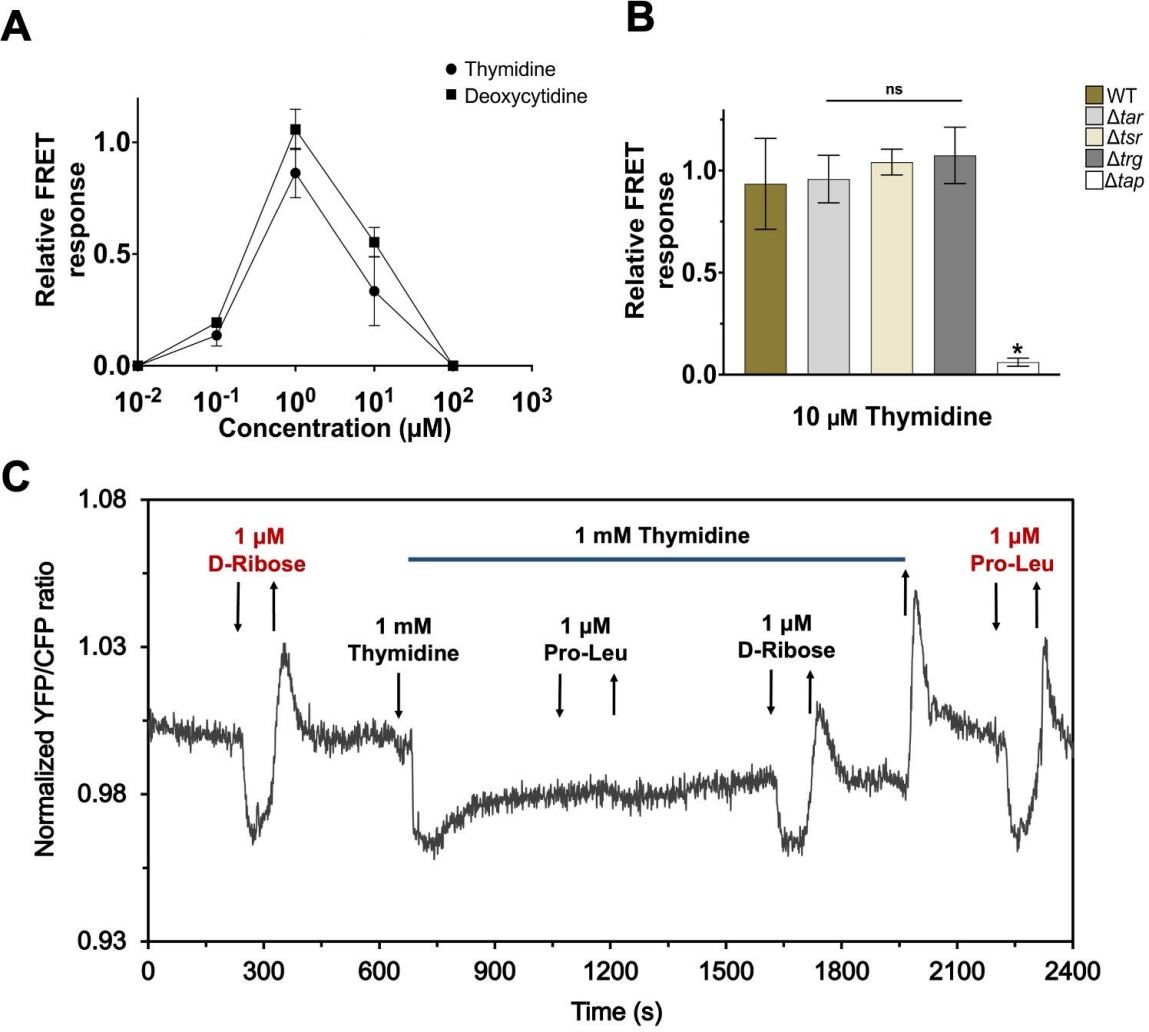
Mechanism of the chemotactic response to pyrimidine deoxyribonucleosides. (**A**) FRET measurement of the dynamic range of the chemotactic response to deoxyribonucleosides. Concentration was raised in 10-fold steps, and cells were allowed to adapt prior to each subsequent stimulation (see Fig. S3). The initial response for each step was normalized to the response of buffer-adapted cells towards a saturating stimulus of 100 µM L-aspartate. Experiments were performed in triplicate. Error bars indicate standard deviations. (**B**) Amplitudes of FRET response to 10 µM thymidine for wild-type *E. coli* and indicated chemoreceptor deletion strains. Each bar represents mean response of three biological replicates, normalized to 100 µM L-aspartate or 100 µM proline-leucine (Pro-Leu) (for Δ*tar*). Error bars indicate standard deviations. Statistical significance was calculated using Student’s two-sample *t*-test between wild-type and deletion strain response (ns: non-significant, * *P*-value ≤ 0.05). (**C**) FRET measurements of competition between response to thymidine and proline-leucine. Trg ligand D-ribose (red) was used as control. Buffer-adapted cells were stimulated and adapted to 1 mM thymidine and, post-adaptation, stimulated with 1 mM thymidine combined with 1 µM proline-leucine. Stimulation with 1 µM proline-leucine in the absence of thymidine (red) was tested post-competition experiment.

We therefore determined the dynamic range of the pathway response to deoxycytidine and thymidine as done previously (28), by raising the concentration of ligands in tenfold steps and allowing cells to adapt prior to each subsequent stimulation. We found that both deoxyribonucleosides show an approximately hundredfold range of concentrations over which they elicit the response upon adaptation, with cells becoming insensitive to further increases above 10 µM (Fig. 2A; Fig. S3). This range is very similar to those previously observed for the indirectly binding ligands galactose, ribose, maltose, or dipeptides and much narrower than the dynamic range for aspartate or serine (28). We conclude that pyrimidine deoxyribonucleosides are likely sensed indirectly, with the response being mediated by a periplasmic binding protein.

To identify the chemoreceptor involved in the sensing of pyrimidine deoxyribonucleosides, we tested FRET responses of *E. coli* strains deleted for individual chemoreceptor genes. While Δ*tar*, Δ*tsr,* and Δ*trg* strains responded to thymidine and deoxycytidine comparably to the wild-type strain, the Δ*tap* strain showed no attractant response to these compounds (Fig. 2B; Fig. S4 and S5). The response to thymidine and deoxycytidine was also present in the strain deleted for *ptsI*, encoding the key PTS enzyme EI that is necessary to mediate PTS responses to the chemotaxis system (8) (Fig. S6). These suggest that the observed response to deoxycytidine and thymidine is mediated by Tap. In contrast, the response to uridine was still preserved in all of the tested receptor and *ptsI* deletion strains, including the Δ*tap* strain (Fig. S4 and S6), which indicates a different and unconventional sensing mechanism that remains to be elucidated.

To further confirm the sensing of pyrimidine deoxyribonucleosides by Tap, we performed a competition assay between thymidine and the known ligand of Tap, proline-leucine, in the wild-type cells. Indeed, the presence of 1 mM thymidine in the background completely inhibited the FRET response to proline-leucine, while not affecting the response to ribose that is indirectly sensed by another receptor, Trg (Fig. 2C). The response to thymidine was also weakened by the presence of 1 mM proline-leucine in the background (Fig. S7). Nevertheless, the response does not depend on the dipeptide-binding periplasmic protein DppA that mediates sensing of proline-leucine by Tap (9) (Fig. S8).

The presence of thymidine also suppressed the response to the ribonucleoside cytidine, suggesting that cytidine shares the deoxyribonucleoside sensing mechanism (Fig. S9A and C). In contrast, the response to uridine was unaffected by thymidine, further supporting our conclusion that it relies on a different sensory mechanism (Fig. S9B and C).

### Conclusions

Extracellular nucleobases and nucleosides can be imported by bacteria and used to replenish their nucleotide pools. They can further serve as sources of carbon, nitrogen, and energy (24). Consistent with that, recent studies indicated that chemotaxis to nucleobases and nucleosides may be common among bacteria (19, 21, 22). Surprisingly, however, chemotactic response to these compounds remained little investigated in the model organism *E. coli*. The only previous study on this topic demonstrated that *E. coli* exhibits a Tap-mediated chemoattraction to millimolar concentrations of pyrimidine nucleobases in a capillary assay (25). However, the concentration threshold of this response was much higher compared with the high-affinity attractants in the same assay (5, 7), raising a question of whether nucleobases are indeed the specific pyrimidine derivatives sensed as attractants by *E. coli*.

Here, we could show that Tap mediates much more sensitive responses to pyrimidine deoxyribonucleosides than to pyrimidine nucleobases. Whereas *E. coli* strongly responded to thymidine (deoxythymidine) and deoxycytidine already at sub-micromolar concentrations, no response to cytosine, uracil, or thymine could be observed even at 10 µM. The sub-micromolar range of concentrations at which the response to pyrimidine deoxyribonucleosides is observed is similar to the response threshold for the most potent established *E. coli* chemoattractants (28). Pyrimidine deoxyribonucleosides appear to be even stronger effectors for Tap than dipeptides, because the adaptation to thymidine completely abolished the response to the dipeptide proline-leucine, whereas the adaptation to proline-leucine only weakened the response to thymidine. These results suggest that deoxyribonucleosides are the primary pyrimidine chemoattractants sensed by Tap, whereas the previously reported response to high concentrations of nucleobases (25) may be a by-product of such deoxyribonucleoside sensing. The lower-affinity response to the ribonucleoside cytidine is also apparently mediated by the same mechanism. In contrast, the response to uridine is apparently Tap-independent and must rely on a different mechanism.

Similar to dipeptides, pyrimidine deoxyribonucleosides appear to be sensed by Tap indirectly. This is suggested by the narrow dynamic range of the response to thymidine or deoxycytidine, which is similar to the dynamic range of the response to dipeptides or sugars that are known to be sensed via the periplasmic binding proteins, but very different from the dynamic range of the response to directly binding amino acids (28). Since the Tap-mediated response to pyrimidine deoxyribonucleosides does not require the dipeptide-binding periplasmic protein DppA, which is again consistent with the previous report for pyrimidine nucleobases (25), it likely relies on another periplasmic protein that remains to be identified.

## MATERIALS AND METHODS

### Bacterial strains and plasmids

*E. coli* W3110 RpoS^+^ (29) was used as the wild-type background for FRET experiments. All chemoreceptor knockout strains were made by P1 transduction from lysates prepared using strains from the Keio collection (30). Km^R^ cassettes were removed by FLP recombination (31). For the expression of the FRET pair CheY-YFP and CheZ-CFP, plasmid pVS88 inducible by isopropyl-β-D-thiogalactoside (IPTG) (32) was transformed into respective *E. coli* strains.

### FRET assays

The FRET measurements were performed as previously described (22, 26, 27, 32). Briefly, *E. coli* cells were grown in tryptone broth (TB) media (1% tryptone, 0.5% NaCl), supplemented with 100 mg/mL ampicillin and 50 µM IPTG at 34°C and 275 r.p.m. Cells were harvested at OD_600_ of 0.6 by centrifugation (4,000×*g* for 5  min), washed with tethering buffer (10  mM KH_2_PO_4_/K_2_HPO_4_, 0.1  mM EDTA, 1  µM methionine, 10 mM sodium lactate, pH 7), and stored at 4°C for 30  min. For microscopy, the cells were attached to the poly-lysine-coated coverslips for 10 min and mounted into a flow chamber that was maintained under constant flow of 0.3 mL/min of tethering buffer using a syringe pump (Harvard Apparatus) that was also used to add or remove compounds of interest (Table S1). FRET measurements were performed on an upright fluorescence microscope (Zeiss AxioImager.Z1) equipped with photon counters (Hamamatsu). CFP fluorescence was excited at 436/20 nm through a 455-nm dichroic mirror by a 75 W Xenon lamp. To detect CFP and YFP emissions, 480/40 nm band pass and 520 nm long pass emission filters were used, respectively. Fluorescence of a monolayer of 300–500 cells was continuously recorded in the cyan and yellow channels using photon counters with a 1.0-s integration time. The fluorescence signals were analyzed, as described previously (26). Gradual drift in the baseline of the YFP/CFP ratio measurements was detrended by calculating linear fit to the data using linear regression in GraphPad Prism. The data were normalized by ratio calculated from the respective fit and used for plotting in all figures and for the calculation of FRET responses.

## Data Availability

All data are present in the manuscript and/or supplemental information. Source data will be made available upon request.

## SUPPLEMENTAL MATERIAL

Supplemental material spectrum.02048-25-s0001.pdf Fig. S1 to S9; Table S1.

ASM does not own the copyrights to Supplemental Material that may be linked to, or accessed through, an article. The authors have granted ASM a non-exclusive, world-wide license to publish the Supplemental Material files. Please contact the corresponding author directly for reuse.

## References

[1] Bi S, Sourjik V. 2018. Stimulus sensing and signal processing in bacterial chemotaxis. Curr Opin Microbiol 45:22–29. doi:10.1016/j.mib.2018.02.002 29459288

[2] Colin R, Ni B, Laganenka L, Sourjik V. 2021. Multiple functions of flagellar motility and chemotaxis in bacterial physiology. FEMS Microbiol Rev 45:fuab038. doi:10.1093/femsre/fuab038 34227665 PMC8632791

[3] Keegstra JM, Carrara F, Stocker R. 2022. The ecological roles of bacterial chemotaxis. Nat Rev Microbiol 20:491–504. doi:10.1038/s41579-022-00709-w 35292761

[4] Parkinson JS, Hazelbauer GL, Falke JJ. 2015. Signaling and sensory adaptation in Escherichia coli chemoreceptors: 2015 update. Trends Microbiol 23:257–266. doi:10.1016/j.tim.2015.03.003 25834953 PMC4417406

[5] Mesibov R, Adler J. 1972. Chemotaxis toward amino acids in Escherichia coli. J Bacteriol 112:315–326. doi:10.1128/jb.112.1.315-326.1972 4562400 PMC251414

[6] Yang Y, M Pollard A, Höfler C, Poschet G, Wirtz M, Hell R, Sourjik V. 2015. Relation between chemotaxis and consumption of amino acids in bacteria. Mol Microbiol 96:1272–1282. doi:10.1111/mmi.13006 25807888 PMC5008178

[7] Adler J, Hazelbauer GL, Dahl MM. 1973. Chemotaxis toward sugars in Escherichia coli. J Bacteriol 115:824–847. doi:10.1128/jb.115.3.824-847.1973 4580570 PMC246327

[8] Somavanshi R, Ghosh B, Sourjik V. 2016. Sugar influx sensing by the phosphotransferase system of Escherichia coli. PLoS Biol 14:e2000074. doi:10.1371/journal.pbio.2000074 27557415 PMC4996493

[9] Manson MD, Blank V, Brade G, Higgins CF. 1986. Peptide chemotaxis in E. coli involves the Tap signal transducer and the dipeptide permease. Nature 321:253–256. doi:10.1038/321253a0 3520334

[10] Bansal T, Englert D, Lee J, Hegde M, Wood TK, Jayaraman A. 2007. Differential effects of epinephrine, norepinephrine, and indole on Escherichia coli O157:H7 chemotaxis, colonization, and gene expression. Infect Immun 75:4597–4607. doi:10.1128/IAI.00630-07 17591798 PMC1951185

[11] Song S, Wood TK. 2021. The primary physiological roles of autoinducer 2 in Escherichia coli are chemotaxis and biofilm formation. Microorganisms 9:386. doi:10.3390/microorganisms9020386 33672862 PMC7918475

[12] Yang J, Chawla R, Rhee KY, Gupta R, Manson MD, Jayaraman A, Lele PP. 2020. Biphasic chemotaxis of Escherichia coli to the microbiota metabolite indole. Proc Natl Acad Sci USA 117:6114–6120. doi:10.1073/pnas.1916974117 32123098 PMC7084101

[13] Hegde M, Englert DL, Schrock S, Cohn WB, Vogt C, Wood TK, Manson MD, Jayaraman A. 2011. Chemotaxis to the quorum-sensing signal AI-2 requires the Tsr chemoreceptor and the periplasmic LsrB AI-2-binding protein. J Bacteriol 193:768–773. doi:10.1128/JB.01196-10 21097621 PMC3021223

[14] Laganenka L, Colin R, Sourjik V. 2016. Chemotaxis towards autoinducer 2 mediates autoaggregation in Escherichia coli. Nat Commun 7:12984. doi:10.1038/ncomms12984 27687245 PMC5056481

[15] Lopes JG, Sourjik V. 2018. Chemotaxis of Escherichia coli to major hormones and polyamines present in human gut. ISME J 12:2736–2747. doi:10.1038/s41396-018-0227-5 29995838 PMC6194112

[16] Pasupuleti S, Sule N, Cohn WB, MacKenzie DS, Jayaraman A, Manson MD. 2014. Chemotaxis of Escherichia coli to norepinephrine (NE) requires conversion of NE to 3,4-dihydroxymandelic acid. J Bacteriol 196:3992–4000. doi:10.1128/JB.02065-14 25182492 PMC4248876

[17] Kondoh H, Ball CB, Adler J. 1979. Identification of a methyl-accepting chemotaxis protein for the ribose and galactose chemoreceptors of Escherichia coli. Proc Natl Acad Sci USA 76:260–264. doi:10.1073/pnas.76.1.260 370826 PMC382918

[18] Bi S, Jin F, Sourjik V. 2018. Inverted signaling by bacterial chemotaxis receptors. Nat Commun 9:2927. doi:10.1038/s41467-018-05335-w 30050034 PMC6062612

[19] Matilla MA, Gavira JA, Krell T. 2023. Accessing nutrients as the primary benefit arising from chemotaxis. Curr Opin Microbiol 75:102358. doi:10.1016/j.mib.2023.102358 37459734

[20] Fernández M, Morel B, Corral-Lugo A, Krell T. 2016. Identification of a chemoreceptor that specifically mediates chemotaxis toward metabolizable purine derivatives. Mol Microbiol 99:34–42. doi:10.1111/mmi.13215 26355499

[21] Monteagudo-Cascales E, Gumerov VM, Fernández M, Matilla MA, Gavira JA, Zhulin IB, Krell T. 2024. Ubiquitous purine sensor modulates diverse signal transduction pathways in bacteria. Nat Commun 15:5867. doi:10.1038/s41467-024-50275-3 38997289 PMC11245519

[22] Xu W, Cerna-Vargas JP, Tajuelo A, Lozano-Montoya A, Kivoloka M, Krink N, Monteagudo-Cascales E, Matilla MA, Krell T, Sourjik V. 2023. Systematic mapping of chemoreceptor specificities for Pseudomonas aeruginosa. mBio 14:e0209923. doi:10.1128/mbio.02099-23 37791891 PMC10653921

[23] Liu X, Wood PL, Parales JV, Parales RE. 2009. Chemotaxis to pyrimidines and identification of a cytosine chemoreceptor in Pseudomonas putida. J Bacteriol 191:2909–2916. doi:10.1128/JB.01708-08 19251854 PMC2681813

[24] Jensen KF, Dandanell G, Hove-Jensen B, WillemoËs M. 2008. Nucleotides, nucleosides, and nucleobases. EcoSal Plus 3. doi:10.1128/ecosalplus.3.6.2 26443734

[25] Liu X, Parales RE. 2008. Chemotaxis of Escherichia coli to pyrimidines: a new role for the signal transducer tap. J Bacteriol 190:972–979. doi:10.1128/JB.01590-07 18065551 PMC2223585

[26] Sourjik V, Vaknin A, Shimizu TS, Berg HC. 2007. In vivo measurement by FRET of pathway activity in bacterial chemotaxis. Methods Enzymol 423:365–391. doi:10.1016/S0076-6879(07)23017-4 17609141

[27] Sourjik V, Berg HC. 2002. Receptor sensitivity in bacterial chemotaxis. Proc Natl Acad Sci USA 99:123–127. doi:10.1073/pnas.011589998 11742065 PMC117525

[28] Neumann S, Hansen CH, Wingreen NS, Sourjik V. 2010. Differences in signalling by directly and indirectly binding ligands in bacterial chemotaxis. EMBO J 29:3484–3495. doi:10.1038/emboj.2010.224 20834231 PMC2964171

[29] Serra DO, Richter AM, Klauck G, Mika F, Hengge R. 2013. Microanatomy at cellular resolution and spatial order of physiological differentiation in a bacterial biofilm. MBio 4:e00103-13. doi:10.1128/mBio.00103-13 23512962 PMC3604763

[30] Baba T, Ara T, Hasegawa M, Takai Y, Okumura Y, Baba M, Datsenko KA, Tomita M, Wanner BL, Mori H. 2006. Construction of Escherichia coli K-12 in-frame, single-gene knockout mutants: the Keio collection. Mol Syst Biol 2:0008. doi:10.1038/msb4100050 PMC1681482 16738554

[31] Datsenko KA, Wanner BL. 2000. One-step inactivation of chromosomal genes in Escherichia coli K-12 using PCR products. Proc Natl Acad Sci USA 97:6640–6645. doi:10.1073/pnas.120163297 10829079 PMC18686

[32] Sourjik V, Berg HC. 2004. Functional interactions between receptors in bacterial chemotaxis. Nature 428:437–441. doi:10.1038/nature02406 15042093

