# Supplementary material for: Response of Escherichia coli chemotaxis pathway to pyrimidine deoxyribonucleosides
Source: Microbiol Spectr. 2025 Oct 14;13(11):e02048-25. doi: 10.1128/spectrum.02048-25 (PMC12584664; doi:10.1128/spectrum.02048-25)
Supplement: Supplemental material — Fig. S1 to S9; Table S1. [file spectrum.02048-25-s0001.pdf]

Supplemental Data

Response of *Escherichia coli* chemotaxis pathway to pyrimidine deoxyribonucleosides

Malay Shah[a], Wenhao Xu[a], and Victor Sourjik[a#]

[a]Max Planck Institute for Terrestrial Microbiology & Center for Synthetic Microbiology (SYNMIKRO), Marburg, Germany.

[#]Address correspondence to Victor Sourjik, victor.sourjik@mpi-marburg.mpg.de

Supplementary figures

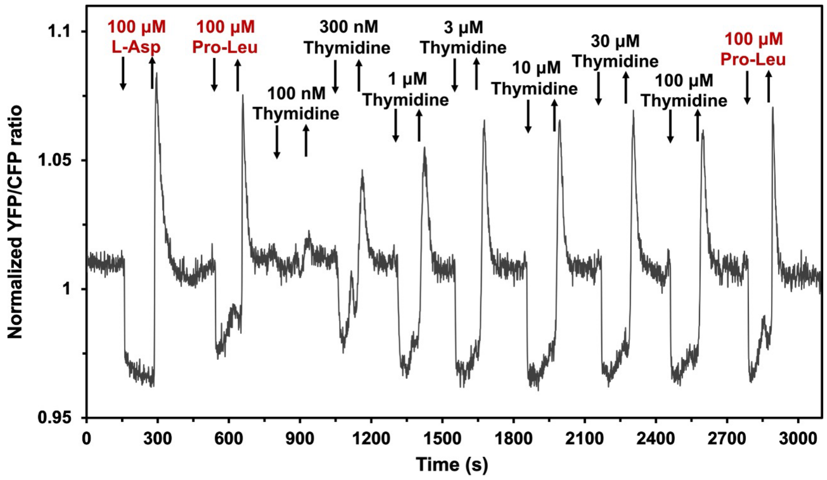

FIG S1 FRET measurement of the chemotaxis pathway response to different concentrations of thymidine. Measurements were performed as in Figure 1. Saturating stimulation with 100 μM L-aspartate (L-Asp; red) was used as a control. Response to 100 μM proline-leucine (Pro-Leu; red) was also measured as positive control for the chemotactic response to dipeptides.

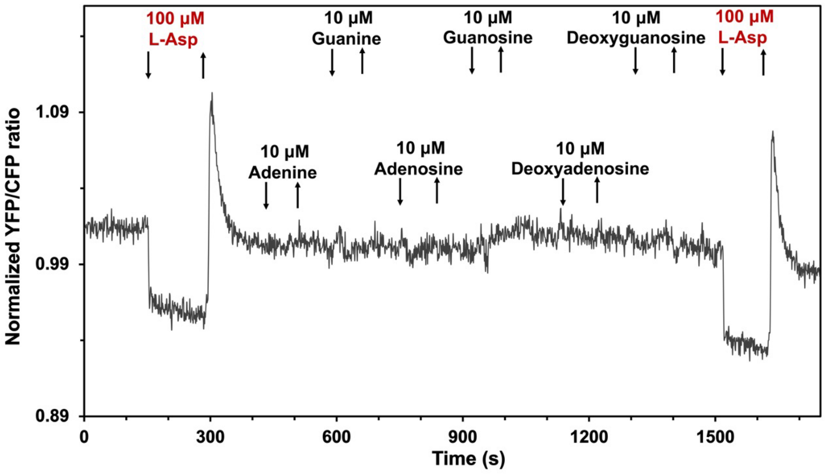

FIG S2 FRET measurement of the chemotaxis pathway response to purine nucleosides and nucleobases. Measurements were performed as in Figure 1. Saturating stimulation with 100 µM L-aspartate (L-Asp; red) was used as a control.

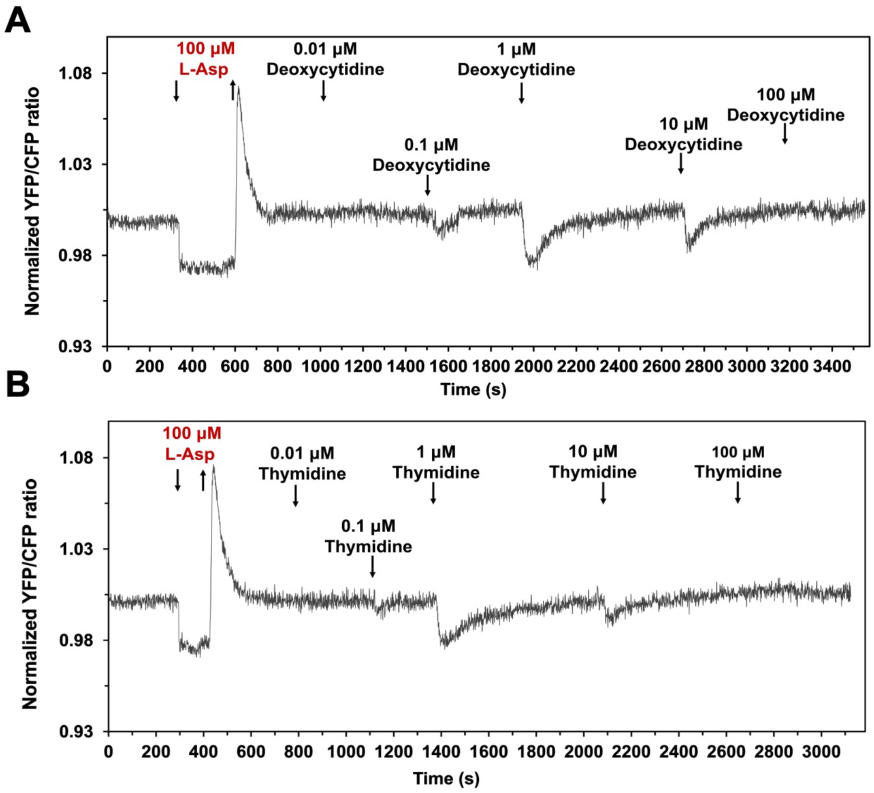

FIG S3 Measurement of the dynamic range of the chemotactic response to deoxyribonucleosides. Concentration of deoxycytidine (**A**) or thymidine (**B**) was raised in 10-fold steps, and cells were allowed to adapt prior to each subsequent stimulation. Saturating stimulation with 100 µM L-aspartate (L-Asp; red) was used as a control. Quantification of these experiments is shown in Figure 2A.

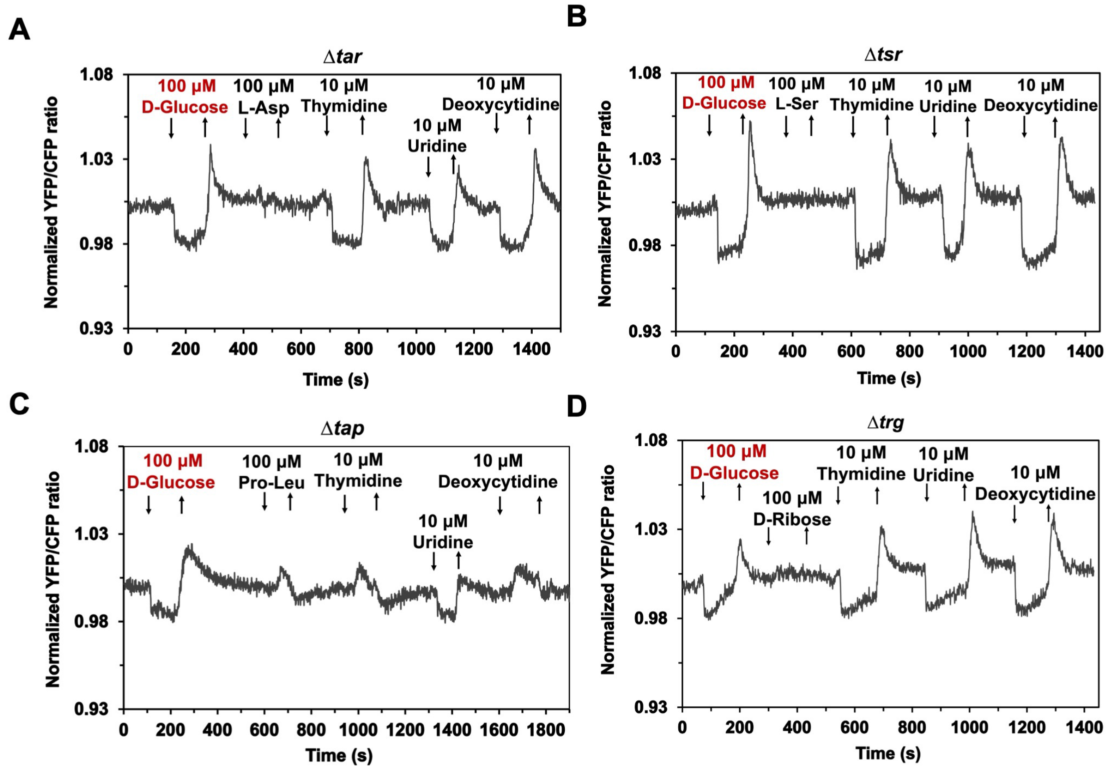

FIG S4 Responses of Δ*tar* (**A**), Δ*tsr* (**B**), Δ*tap* (**C**), and Δ*trg* (**D**) chemoreceptor deletion strains to deoxyribonucleosides (black) and control compounds (red) at indicated concentrations. Measurements were performed as in Figure 1. Canonical ligands of individual receptors were measured for each receptor mutant at indicated concentrations. No response was detected for L-asparate (L-Asp) in Δ*tar*, L-serine (L-Ser) in Δ*tsr*, proline-leucine (Pro-Leu) in Δ*tap* and D-ribose in Δ*trg*. D-glucose sensed as an attractant via the PTS was used as a positive control (red). Quantification of these experiments is shown in Figure 2B and Figure S5. Minor increase in the YFP/CFP ratio upon the addition of proline-leucine or deoxyribonucleosides in Δ*tap* is likely unspecific or indicates a weak repellent response.

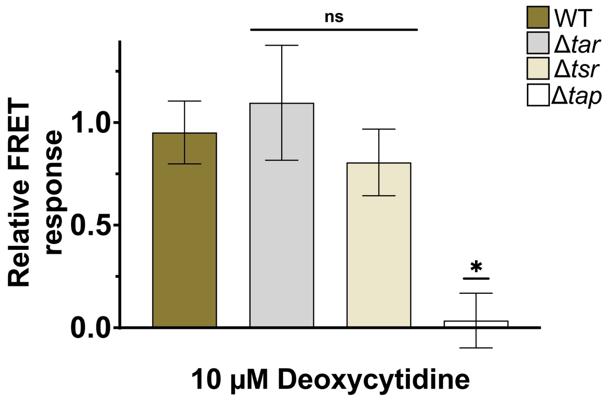

FIG S5 Dependence of the response to deoxycytidine on Tap (B). Measurements were performed as shown in Figure S4 and quantified as described in Figure 2B. Statistical significance was calculated using Student's two-sample *t*-test between wild- type and knockout (ns: non-significant, * *P*-value ≤0.05).

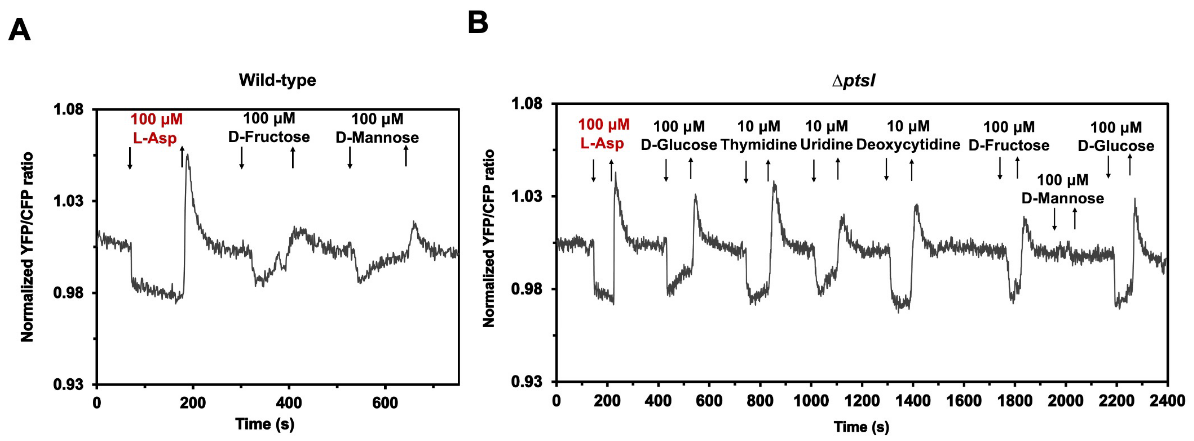

FIG S6 Responses of the wild-type (**A**) and of Δ*ptsI* (**B**) chemoreceptor deletion strain to deoxyribonucleosides (black) at indicated concentrations. Measurements were performed as in Figure 1. Saturating stimulation with 100 µM L-aspartate (L-Asp; red) was used as a control.

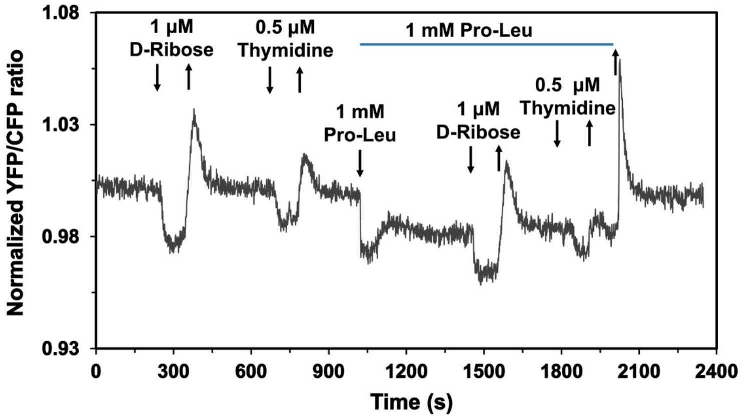

FIG S7 FRET measurements of competition between response to proline-leucine and thymidine. Trg ligand ribose was used as control. Buffer adapted cells were stimulated and adapted to 1 mM proline-leucine (Pro-Leu) and, post adaptation, stimulated with 0.5 µM thymidine. Stimulation with 0.5 µM thymidine in the absence of proline-leucine was tested as a control before the competition experiment. Stimulation with the Trg-specific attractant D-ribose was used as a positive control.

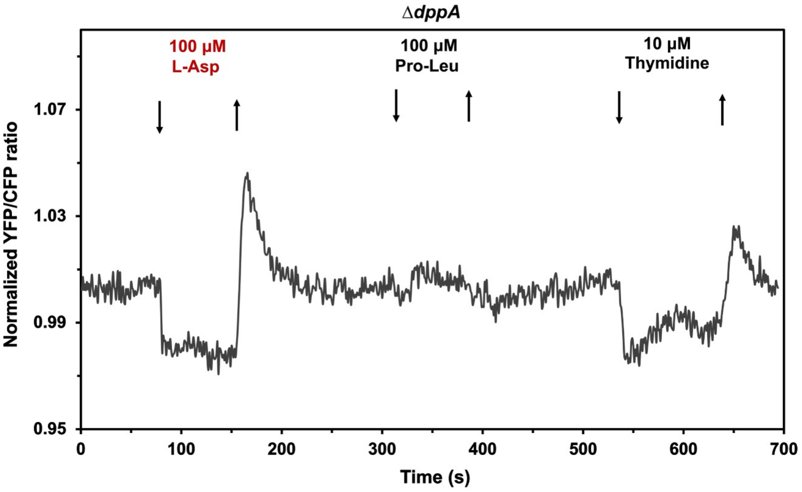

FIG S8 Response of Δ*dppA* chemoreceptor deletion strain to thymidine. No response to the dipeptide ligand proline-leucine (Pro-Leu) was observed in Δ*dppA*. Saturating stimulation with 100 µM L-aspartate (L-Asp; red) was used as a control. Measurements were performed as in Figure 1.

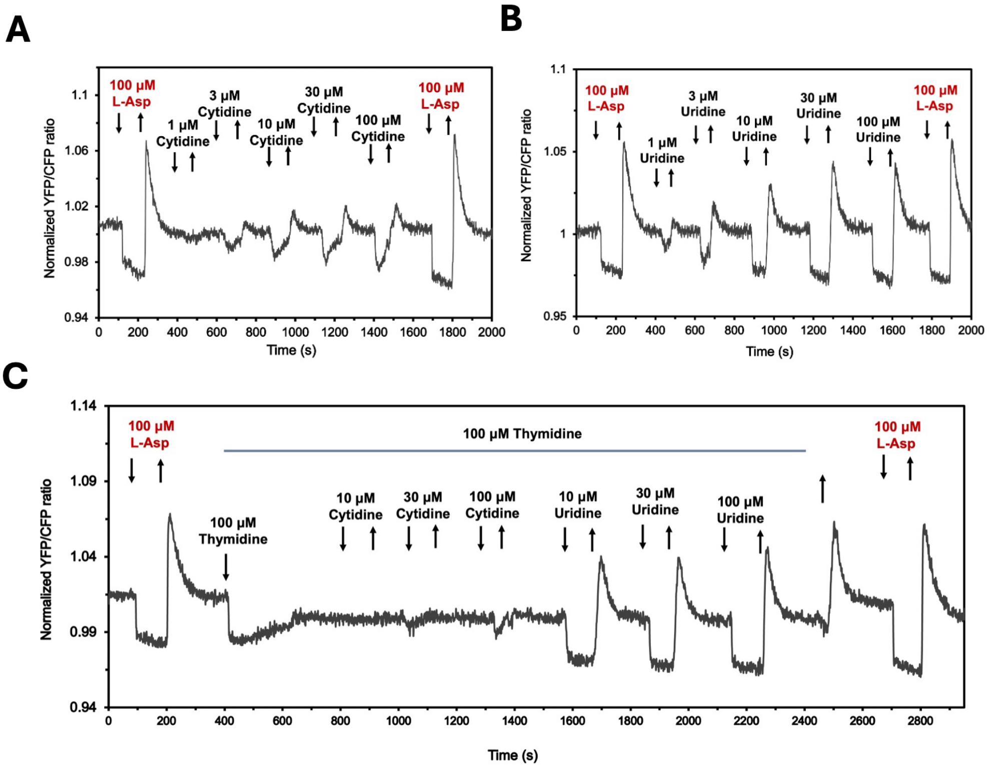

FIG S9 FRET measurements of the chemotaxis pathway response to different concentrations of cytidine (A) and uridine (B). Measurements were performed as in Figure 1. Saturating stimulation with 100 μM L-aspartate (L-Asp; red) was used as a control. (C) Response competition between thymidine and cytidine or uridine. Measurements were performed as in Figure 2C. Buffer adapted cells were stimulated and adapted to 100 μM thymidine and, post adaptation, stimulated with different concentrations of cytidine and uridine in the presence of 100 μM thymidine. Saturating stimulation with 100 μM L-aspartate (L-Asp; red) was used as a control.

Table S1: List of chemical compounds used in the study

Compound	Company	Cas number	Purity
L-Aspartate	Roth	56-84-8	≥99% (HPLC)
Cytosine	Roth	7295.1	≥99 %, for biochemistry
Thymine	Sigma	65-71-4	≥99%, synthetic powder
Cytidine	Sigma	65-46-3	≥99%
Uridine	Sigma	58-96-8	≥99%, synthetic powder
Thymidine	Sigma	50-89-5	≥99%
Deoxycytidine	Sigma	951-77-9	≥99% (HPLC), synthetic powder
D-Ribose	Sigma	50-69-1	≥99% (GC)
Proline-leucine	Sigma	52899-07-7	≥98% (TLC)
Adenine	Roth	73-24-5	≥98%, for biochemistry
Guanine	Roth	73-40-5	≥97%, for biochemistry
Adenosine	Roth	58-61-7	≥99% (HPLC)
Guanosine	Sigma	118-00-3	≥98%
Deoxyadenosine	Sigma	16373-93-6	≥99%, synthetic powder
Deoxyguanosine	Sigma	312693-72-4	99-100%, synthetic powder
L-Serine	Roth	56-45-1	≥99%
D-Fructose	Sigma	57-48-7	≥99% (HPLC)
D-Mannose	Sigma	3458-28-4	≥99%
D-Glucose	Roth	50-99-7	p.a., ACS, anhydrous
